# A Comprehensive Health Screening Program Reveals the Prevalence of and Risk Factors for Age-Related Macular Degeneration: A Cross-Sectional Analysis

**DOI:** 10.3390/biomedicines12122681

**Published:** 2024-11-25

**Authors:** Dae Joong Ma, Baek-Lok Oh, Eunoo Bak, Jin-Soo Kim, Jinho Lee, Hyuk Jin Choi

**Affiliations:** 1 Department of Ophthalmology, Seoul National University College of Medicine, Seoul 03080, Republic of Korea; daejoongma@hallym.or.kr (D.J.M.);; 2 Department of Ophthalmology, Hallym University Kangnam Sacred Heart Hospital, Seoul 07441, Republic of Korea; 3 Genome Insight, Inc., Daejeon 34051, Republic of Korea; 4 Department of Ophthalmology, Uijeongbu Eulji Medical Center, Eulji University School of Medicine, Uijeongbu 11759, Republic of Korea; 5 Department of Ophthalmology, Chungnam National University School of Medicine, Daejeon 34134, Republic of Korea; 6 Hana Seoul Eye Clinic, Bucheon 14537, Republic of Korea; 7 Department of Ophthalmology, Seoul National University Hospital Healthcare System Gangnam Center, Seoul 06236, Republic of Korea

**Keywords:** age-related macular degeneration, health screening center, prevalence, risk factor

## Abstract

**Background/Objectives**: We investigated the prevalence of age-related macular degeneration (AMD) and associated risk factors in Korean subjects who underwent comprehensive health screening examinations. **Methods**: This single health screening center-based cross-sectional study included a total of 73,574 consecutive participants older than 30 years who underwent a health screening examination, including fundus photography, between October 2003 and December 2010. Weighted prevalence and risk factors for AMD were evaluated. Logistic regression was used to identify AMD risk factors. **Results**: The weighted prevalence of AMD was 15.42%, with a prevalence of 3.34% among people in their 30s. Advanced age significantly increased the risk for both early/intermediate AMD (*p* < 0.001 across the age groups of 40, 50, 60, and 70+ years) and advanced AMD (*p* <0.001 for the age groups of 60 and 70+ years). The male sex was strongly associated with an increased risk of both early/intermediate and advanced AMD (*p* < 0.001 for both). Retinal arteriosclerosis, whether low- or high-grade, was linked to early/intermediate AMD (*p* < 0.001 for both grades), whereas only high-grade arteriosclerosis was linked to advanced AMD (*p* < 0.001). Additionally, hypertension (*p* < 0.001), the hepatitis B carrier status (*p* < 0.001), elevated mean corpuscular volume (*p* < 0.001), and lower serum uric acid levels (*p* = 0.014) were associated with early/intermediate AMD. Higher education levels protected against early/intermediate AMD (*p* = 0.004 for high school graduates, *p* < 0.001 for ≥college graduates). Higher serum inorganic phosphate levels (*p* = 0.002) and lower total serum ALB levels (*p* = 0.005) were significant risk factors for advanced AMD. **Conclusions**: Korean individuals as young as 30 years old are at risk of AMD. This study newly identified associations between retinal arteriosclerosis and both early/intermediate and advanced AMD, as well as associations between serum inorganic phosphate levels and total ALB levels with advanced AMD.

## 1. Introduction

**Figure 1:**
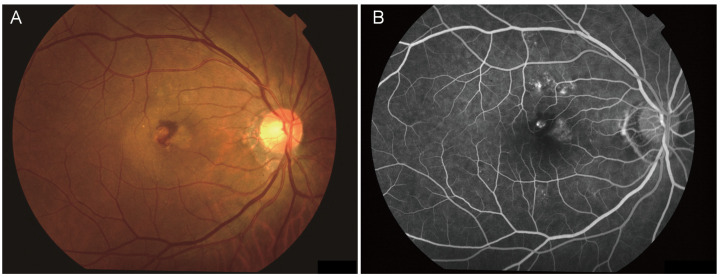
Fundus photograph (**A**) and fluorescein angiogram (**B**) of a 53-year-old Korean male diagnosed with exudative age-related macular degeneration.

Age-related macular degeneration (AMD) is a disease that affects the macula, leading to a gradual decline in central vision (Figure 1) [1]. AMD currently affects approximately 20 million people in the United States and 196 million people worldwide [2]. As one of the primary causes of severe vision impairment in older adults, AMD is projected to impact approximately 288 million people globally by 2040.

The pathologic mechanisms underlying the development and progression of AMD primarily involve the impairment and degeneration of the retinal pigment epithelium (RPE) [3]. RPE cells play a critical role in maintaining retinal structure and function, as they contribute to the integrity of the outer blood–retina barrier, secrete essential retinal growth factors, and participate in the visual cycle and local immune responses [4]. Therefore, impairment in RPE function or anatomy can lead to vision-affecting diseases. Due to their active oxidative metabolism, RPE cells are exposed to high levels of reactive oxygen species [5]. Additionally, an increase in oxidative stress and dysfunction of antioxidant defense mechanisms lead to structural degeneration of the choriocapillaris, reducing blood flow to the RPE and photoreceptors [6]. This impaired circulation hampers the clearance of lipids, proteins, and other waste materials, which then accumulate as drusen. These deposits trigger remodeling of the extracellular matrix and initiate an inflammatory response. Due to the complex interplay of these processes, AMD can progress to either atrophic or neovascular stages [7].

Identifying the prevalence and risk factors for AMD is of paramount importance, as it enables the early detection of at-risk individuals, aids in prognostic predictions, and guides treatment strategies. Furthermore, addressing modifiable risk factors, such as quitting smoking, maintaining a healthy weight, adopting a diet rich in omega-3 long-chain polyunsaturated fatty acids, and effectively managing blood pressure and lipid levels, can partially prevent AMD [8]. However, numerous studies have highlighted variations in AMD prevalence and risk factors across different racial groups [9]. Moreover, variations in AMD characteristics among individuals across different Asian countries are noteworthy. Hence, specific research on the prevalence of and risk factors for AMD based on racial and national differences is needed.

Hospital-based studies typically involve patients with severe conditions requiring treatment, focusing primarily on the effectiveness of treatment strategies. In contrast, health screening center-based studies cover broader populations, including both asymptomatic individuals and patients with milder conditions. This approach would provide more comprehensive information on risk factors and early detection methods for a range of diseases. In addition, health screening-based studies often involve a wide array of diagnostic tests and clinical evaluations, allowing for a detailed assessment of multiple organ systems and an evaluation of multiorgan interactions, unlike population-based studies.

Two large population-based studies utilizing data from the Korea National Health and Nutrition Examination Survey (KNHANES) identified risk factors for AMD in the Korean population. Nevertheless, the findings were derived from a limited set of diagnostic tests and clinical evaluations, which constrains the ability to pursue further investigations and hinders the potential to expand the research scope comprehensively.

Accordingly, we conducted epidemiologic analyses and investigated the factors associated with AMD using baseline data from the Gangnam Eye Cohort. This large retrospective cohort was composed of Korean participants who underwent comprehensive health screening examinations, including fundus photography, and were followed for more than 10 years.

The results of this research may elucidate the complex interplay of factors contributing to the development and progression of AMD, personalize prevention strategies, and provide a deeper understanding of the underlying mechanisms of the disease. The results of this study will be reviewed alongside other extensive health screening programs that assess various organ systems, with the goal of broadening the scope of research thoroughly.

## 2. Materials and Methods

### 2.1. Participant Population

The Gangnam Eye Cohort consisted of Korean individuals who underwent an organized health screening program, including fundus photography, at the Seoul National University Hospital Healthcare System Gangnam Center between October 2003 and December 2023. Among them, baseline data from October 2003 and December 2010 were utilized, and only subjects aged 30 years or older were included in this study. The study was approved by the Seoul National University Hospital Institutional Review Board (IRB No. H-1906-141-1043). All procedures adhered to the Declaration of Helsinki, and the requirement for patient consent was waived because of the retrospective design of the study.

### 2.2. Comprehensive Health Screening Examinations and Definition of Variables

The health interview survey included standardized questionnaires regarding sociodemographic factors, health-related lifestyle behaviors, and past and current medical conditions. We considered the sociodemographic factors as follows: stratified age (30 to 39 years old, 40 to 49 years old, 50 to 59 years old, 60 to 69 years old, and 70 years old or older), monthly household income (<KRW 3,000,000, ≥KRW 3,000,000 and <KRW 5,000,000, ≥KRW 5,000,000 and <KRW 10,000,000, and ≥KRW 10,000,000), and education level (did not complete middle school, middle school graduates, high school graduates, and college/university graduates or higher). The following factors were included in the health-related lifestyle behaviors: smoking status (never smoked, former smokers, and current smokers), drinking status (never drinkers, former drinkers, and current drinkers), and physical activity level. The physical activity level was classified into the following categories: active lifestyle (≥2 h of exercise a week of at least moderate intensity) or sedentary lifestyle (<2 h of exercise a week of at least moderate intensity).

The following information was collected on past and current medical conditions: diabetes mellitus (DM), systemic hypertension (HTN), dyslipidemia, cardiovascular diseases, cerebrovascular disease, and cancer. Patients who met any of the following criteria were considered to have the associated systemic disease. For DM, patients indicated that they (1) had DM, (2) used antidiabetic medications, (3) had a fasting glucose level ≥ 126 mg/dL, or (4) had a glycosylated hemoglobin (HbA1c) level ≥ 6.5%. For HTN, patients indicated that they (1) had HTN, (2) used antihypertensive medication, or (3) had a systolic blood pressure (BP) ≥ 140 mmHg and/or diastolic BP ≥ 90 mmHg. For dyslipidemia, patients indicated that they (1) had dyslipidemia, (2) used anti-dyslipidemic medications, (3) had a total cholesterol concentration ≥ 240 mg/dL or met one or more of the following criteria: triglyceride (TG) levels ≥ 200 mg/dL, low-density lipoprotein (LDL) cholesterol levels ≥ 160 mg/dL, or high-density lipoprotein (HDL) cholesterol levels < 40 mg/dL.

Blood samples were collected from the antecubital vein after a minimum fasting period of 10 h. The comprehensive blood tests included serum levels of corrected calcium, inorganic phosphate, blood urea nitrogen (BUN), creatinine, aspartate transaminase (AST), alanine transaminase (ALT), alkaline phosphatase (ALP), gamma-glutamyl transferase (GGT), total bilirubin, uric acid (UA), total cholesterol, HDL cholesterol, LDL cholesterol, TG, total albumin, high-sensitivity C-reactive protein (Hs-CRP), hemoglobin (Hb), and HbA1c; white blood cell (WBC) counts; platelet counts; mean corpuscular volume (MCV); serum surface antigen of hepatitis B virus (HBsAg); antibodies against the hepatitis C virus (anti-HCV); and antibodies against *Helicobacter pylori*.

Nonmydriatic fundus photography was performed with a 45° field-angle digital fundus camera (CR6-45NW; Canon, Inc., Utsunomiya, Japan) in a dark room.

### 2.3. Diagnosis of Age-Related Macular Degeneration and Retinal Arteriosclerosis

AMD was graded using the grading protocol of the original classification used in the Age-Related Eye Disease Study (AREDS) [10,11]. Briefly, fundus photographs were used to define early AMD if small drusen (<63 µm) with a total area ≥ 125 µm in diameter; at least one intermediate drusen (63 to 124 µm in diameter); any hypopigmentation; or hyperpigmentation with a total area ≥ 125 µm in diameter was present. Intermediate AMD was defined if fundus photographs revealed soft indistinct intermediate drusen with a total area ≥ 360 µm in diameter, soft distinct intermediate drusen with a total area ≥ 656 µm in diameter, at least one large drusen (>125 µm in diameter), or geographic atrophy (GA) not involving the center of the fovea. Advanced AMD was defined if fundus photographs showed a GA-involving center of the macula or signs of choroidal neovascularization. Drusen and GA were assessed within 2 disc diameters of the center of the macula, whereas pigment abnormalities were assessed within 1 disc diameter of the center of the macula.

Retinal arteriosclerosis was graded based on the Scheie classification, with slight modifications [12,13]. Briefly, Grade 0 was defined as no abnormalities. Visible broadening of the light reflex from the artery with minimal or no arteriovenous compression was defined as Grade 1. Changes similar to those in Grade 1 but with more prominent or visible signs of arteriovenous crossing were defined as Grade 2. Grade 3 was characterized by the presence of arteries with a “copper wire” appearance and more pronounced arteriovenous compression. Arteries with a “silver wire” appearance and the most severe arteriovenous crossing changes were defined as Grade 4. In the present study, Grade 1 was defined as “low”, and Grade 2 or higher was defined as “high” retinal arteriosclerosis.

### 2.4. Statistical Analyses

Comparisons by AMD grade were conducted using either the independent *t* test or chi-square test, depending on the type of variable. The Cochran–Armitage trend test was conducted to evaluate age-related trends in AMD prevalence.

Descriptive statistics were calculated for the original (non-imputed) dataset to accurately represent the study population. Multiple imputation was utilized to increase the analytical power and address missing covariates in the logistic regression analysis (LRA). The distribution of missing data by variable is presented in Supplementary Materials: Table S1. Twenty imputed complete datasets were generated, and the LRA results from each imputed dataset were pooled together via Rubin’s rules to obtain overall estimates [14]. Multivariate LRAs were corrected using the Benjamini–Hochberg (B–H) correction to reduce the risk of type I error in this study.

Post-stratification weighting adjustment was applied to ensure that the weighted prevalence represented the Korean population as closely as possible [15]. Weighting was applied based on the age and sex distributions in the census data as of 31 December 2006, which coincided with the midpoint of our recruitment phase.

All variables with a significance of *p* < 0.10 in the univariate analysis were incorporated into a multivariate logistic regression model. Adjusted odds ratios (ORs) and 95% confidence intervals (CIs) were calculated to assess the strengths of the relationships. *p* < 0.05 was considered to indicate statistical significance. The analysis was conducted using SPSS 22.0 software for Windows, developed by SPSS, Inc., which is based in Chicago, IL, USA.

## 3. Results

### 3.1. Demographic and Clinical Characteristics

**Figure 2:**
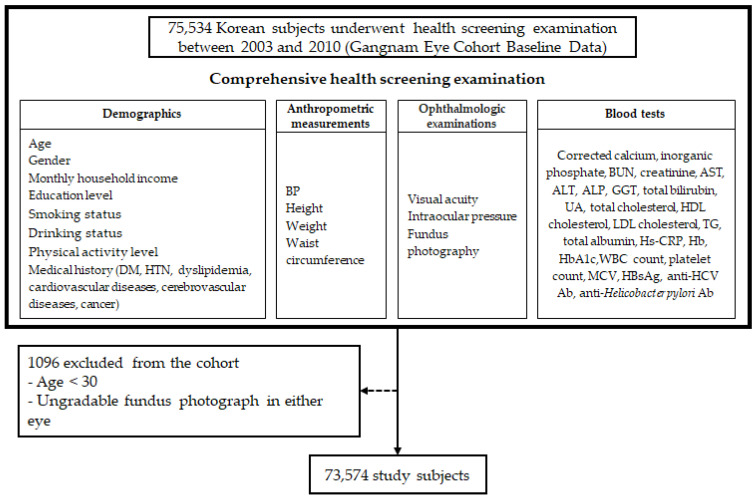
Flowchart of study population selection and inclusion criteria. Ab = antibody; ALP = alkaline phosphatase; ALT = alanine transaminase; AST = aspartate transaminase; BUN = blood urea nitrogen; Cr = creatinine; DM = diabetes mellitus; GGT = gamma-glutamyl transferase; HBsAg = hepatitis B surface antigen; HCV = hepatitis C; Hb = hemoglobin; HbA1c = glycosylated hemoglobin; HDL = high-density lipoprotein; Hs-CRP = high-sensitivity C-reactive protein; MCV = mean corpuscular volume; P = inorganic phosphate; TG = triglyceride; UA = uric acid; WBC = white blood cell.

Between October 2003 and December 2010, a total of 75,534 Korean individuals underwent comprehensive health screening examinations, including fundus photography. A total of 73,574 subjects were included in the present study after excluding 1096 subjects who were younger than 30 years old or had ungradable fundus photographs of either eye (as shown in Figure 2).

The baseline characteristics of the total study population, as well as those of subjects with and without AMD, are summarized in Table 1, which also includes a comparison between subjects with and without AMD. In subjects with AMD, the proportions of patients in the older age groups and males were significantly greater (both, *p* < 0.001), as were the rates of former smokers and those with an active lifestyle (both, *p* < 0.001). Additionally, significantly greater proportions of individuals with AMD had a history of DM, HTN, dyslipidemia, cardiovascular disease, cerebrovascular disease, or cancer (all *p* < 0.001). In terms of socioeconomic factors, subjects with AMD had significantly lower household income and education levels (both *p* < 0.001). Interestingly, women with AMD were shorter and had a significantly greater BMI (both, *p* < 0.001), whereas men with AMD were both shorter and had a lower BMI (*p* <0.001 and *p* = 0.009, respectively).

The serum levels of BUN, creatinine, AST, ALT, ALP, GGT, uric acid, total cholesterol, triglycerides, and Hs-CRP were significantly elevated in the subjects with AMD (all *p* < 0.001). Additionally, the total bilirubin level was also significantly higher (*p* = 0.001). Conversely, the serum levels of HDL and total albumin were significantly lower (both *p* < 0.001). Furthermore, in subjects with AMD, Hb levels, WBC counts, MCVs, and HbA1c levels were significantly elevated (*p* < 0.001, *p* = 0.017, and *p* < 0.001, respectively), whereas platelet counts were significantly reduced (*p* < 0.001). Additionally, rates of HBsAg-positive and anti-HCV antibody-positive statuses were significantly higher among AMD patients (both *p* < 0.001). Finally, the proportion of individuals with high-grade retinal arteriolar sclerosis was significantly elevated in the AMD group (*p* < 0.001).

In addition, the baseline characteristics of the imputed data are presented in Supplementary Materials, Table S2.

### 3.2. Weighted Prevalence of AMD in the Gangnam Eye Cohort

The weighted prevalence of AMD was 15.42% (95% CI, 15.16–15.68%), with 13.72% (95% CI, 13.47–13.97%) for early AMD, 1.31% (95% CI, 1.23–1.39%) for intermediate AMD, and 0.39% (95% CI, 0.35–0.44%) for advanced AMD (Table 2). The weighted prevalence of AMD by age group is presented in Table 2.

The Cochran–Armitage trend test for age groups revealed a statistically significant increasing trend in the prevalence of overall AMD, early AMD, intermediate AMD, and advanced AMD with increasing age (all *p* < 0.001).

**Figure 3:**
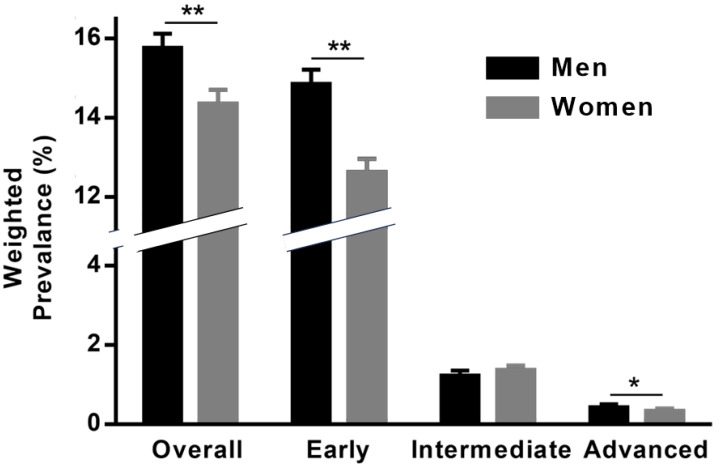
Bar graph showing the sex-specific weighted prevalence of early, intermediate, and advanced age-related macular degeneration in the Gangnam Eye Cohort (2003–2010). ** *p* < 0.01, men vs. women; * *p* < 0.05, men vs. women.

Figure 3 shows that the weighted prevalence of overall AMD was greater in men (15.77%; 95% CI, 15.40–16.13%) than in women (14.36%; 95% CI, 14.01–14.71%; *p* < 0.001). Similarly, the weighted prevalence of early AMD was greater in men (14.86%; 95% CI, 14.49–15.22%) than in women (12.64%; 95% CI, 12.30–12.97%; *p* < 0.001), and the weighted prevalence of advanced AMD was also greater in men (0.44%; 95% CI, 0.37–0.51%) than in women (0.35%; 95% CI, 0.29–0.40%; *p* = 0.044). However, the weighted prevalence of intermediate AMD was similar in men (1.24%; 95% CI, 1.13–1.36%) and women (1.38%; 95% CI, 1.26–1.49%; *p* = 0.112).

### 3.3. Risk Factors for Early/Intermediate AMD

The characteristics of participants with normal and early/intermediate age-related macular degeneration before and after multiple imputation are presented in Table S3, which also includes a comparison between subjects without AMD and early/intermediate AMD.

The results of the LRAs obtained by grouping patients with early/intermediate AMD are summarized in Table 3, with the reference category being individuals without AMD. According to the multivariate LRA, early/intermediate AMD was positively associated with all age groups ≥ 40 years (all B–H-corrected *p* < 0.001), male sex (B–H-corrected *p* < 0.001), a history of HTN (B–H-corrected *p* < 0.001), MCV (B–H-corrected *p* = 0.001), and serum HBsAg positivity (B–H-corrected *p* < 0.001). Similarly, both low and high retinal arteriosclerosis levels were positively associated with early/intermediate AMD in the multivariate LRA (all B–H-corrected *p* < 0.001). In contrast, education levels of high school graduates and college/university graduates or higher (B–H-corrected *p* = 0.004 and <0.001, respectively) and serum UA levels (B–H-corrected *p* = 0.014) were negatively associated with early/intermediate AMD in the multivariate LRA. The results from the regression analysis of the original (non-imputed) data are presented in Supplementary Materials, Table S4.

### 3.4. Risk Factors for Advanced AMD

The characteristics of participants with normal and advanced age-related macular degeneration before and after multiple imputation are presented in Table S5, which also includes a comparison between subjects without AMD and advanced AMD.

Table 4 presents the results of the LRAs for patients with advanced AMD, with individuals without AMD serving as the reference category. According to the multivariate LRA, advanced AMD was positively associated with all age groups 60 years and older (60–69 years and 70 years and older; all B–H-corrected *p* < 0.001), male sex (B–H-corrected *p* < 0.001), and the serum concentration of inorganic phosphate (B–H-corrected *p* = 0.002). Additionally, a high level of retinal arteriosclerosis was positively associated with advanced AMD according to the multivariate LRA (B–H-corrected *p* < 0.001). Conversely, the serum level of total albumin (B–H-corrected *p* = 0.005) was negatively associated with advanced AMD in the multivariate LRA. The results from the regression analysis of the original (non-imputed) data are presented in Supplementary Materials, Table S6.

## 4. Discussion

In the present study, the weighted prevalence of AMD in Koreans older than 30 years was 15.42%, comprising 13.72% with early AMD, 1.31% with intermediate AMD, and 0.39% with advanced AMD. The weighted prevalence for individuals in their 30s was 3.34%. An older age was positively associated with both early/intermediate and advanced AMD, with ORs of 2.30, 3.86, 5.89, and 9.32 for individuals aged 40, 50, 60, and 70+ years, respectively. For advanced AMD, the ORs increased to 11.88 and 29.21 for those aged 60 and 70+ years, respectively. The male sex was also positively associated, with ORs of 1.83 and 2.88 for early/intermediate and advanced AMD, respectively. Increased retinal arteriosclerosis was positively correlated with AMD, with ORs of 1.35 and 1.34 for low and high arteriosclerosis in patients with early/intermediate AMD, respectively, and an OR of 3.14 for high arteriosclerosis in patients with advanced AMD. In contrast, a history of HTN (OR 1.12), increased MCV (OR 1.01), and the HBV carrier status (OR 1.47) were positively associated with only early/intermediate AMD. Higher education (ORs of 0.87 and 0.80 for high school and college graduates, respectively) and higher serum UA levels (OR of 0.97) were negatively associated with early/intermediate AMD. Additionally, serum inorganic phosphate levels (OR 1.45) were positively associated with advanced AMD, whereas serum albumin levels (OR 0.30) were negatively associated with advanced AMD. The associations of AMD with retinal arteriosclerosis, serum inorganic phosphate levels, and total albumin levels were established for the first time in this study.

Compared with data from representative Korean population-based studies utilizing KNHANES data [16,17], our findings revealed a higher prevalence of early/intermediate AMD, whereas the prevalence of advanced AMD remained similar. This difference may be attributed to the disparity in the AMD classification schemes applied to each dataset. In studies utilizing KNHANES data, researchers adopted the International Age-Related Maculopathy Epidemiological Study Group’s grading system. This system does not classify pigmentary abnormalities as early AMD in the absence of soft drusen [18]. In contrast, the original AREDS classification adopted in the present study considers pigmentary abnormalities as early AMD, even without soft drusen, which leads to a higher prevalence of early AMD.

Intriguingly, although AMD typically affects elderly individuals, our study revealed AMD features based on fundus photographs in individuals aged 30–40 years, with a prevalence of 3.34%. Most population-based studies on AMD have traditionally focused on individuals older than 40 years [19,20], which may have limited the understanding of AMD in younger age groups. The inclusion of younger subjects aged 30–40 years allows a more comprehensive understanding of the early risk factors for AMD, potentially leading to more effective prevention strategies in this population.

Age is the most potent risk factor for AMD, as evidenced by numerous studies. With respect to early/intermediate AMD [21,22], age groups older than 40 years presented a significantly greater risk than did the reference group aged 30–40 years, and this risk increased linearly with each additional decade of age. Conversely, in the case of advanced AMD, individuals older than 60 years presented a substantially greater risk than did individuals in the reference group aged 30–40 years, with this risk increasing almost exponentially with age.

This study revealed that male sex was a significant risk factor for both early/intermediate and advanced AMD, with a more pronounced association observed for advanced AMD. The relationship between sex and AMD risk varies across different populations. In white populations, most studies have shown a greater risk of AMD in females [19,20], whereas a greater risk has been observed in males in Asian populations [9,23]. The reasons for the sex-related disparities in AMD risk among different races remain unclear. However, polypoidal choroidal vasculopathy, a dominant subtype of advanced AMD in Asians, is predominant in males [24]. This disorder likely contributes to the increased risk of advanced AMD in Asian males. Furthermore, this may also be attributed to the significantly high prevalence of smoking among Asian men [9].

A history of HTN was also positively associated with early/intermediate AMD. This finding aligns with findings from the AREDS study, which also highlighted HTN as a risk factor for the development of large drusen and neovascular AMD [25]. Elevated BP is associated with reduced choroidal blood flow and vascular disturbances, contributing to AMD pathogenesis [26].

Our study revealed that a decreased risk of early/intermediate AMD was associated with higher education levels, although previous studies have reported inconsistent results regarding the link between the education level and AMD risk [25,27]. Importantly, the education level itself is not a direct risk factor but serves as a surrogate for other risk factors associated with an unhealthy lifestyle. In addition, individuals with advanced education are significantly more inclined to pursue white-collar or indoor professions, a pattern consistently highlighted in various studies as being linked to a decreased risk of AMD compared with blue-collar or outdoor occupations [17,28].

The following factors associated with AMD identified in this study warrant significant attention, as they either (1) represent novel findings or (2) have been reported in a notably limited number of previous studies, thereby contributing new insights into the etiology of AMD. In this study, for the first time, we demonstrated that retinal arteriosclerosis, graded using the modified Scheie classification system, is positively associated with both early/intermediate and advanced stages of AMD. Systemic atherosclerosis is known to contribute to AMD development [29,30], potentially through mechanisms such as thickening and stiffening of choroidal vessel walls, which increase blood flow resistance and reduce retinal perfusion, ultimately impairing the RPE [31]. Additionally, elevated hydrostatic pressure in the choroidal vessels can lead to protein and lipid leakage into the retina. These leaks result in the formation of basal deposits within Bruch’s membrane and drusen between its inner surface and the RPE [32]. Large drusen and basal deposits can compromise the RPE, causing pigment clumping or geographic atrophy. Since retinal arteriosclerosis serves as an indicator of systemic atherosclerosis, this finding may help explain its association with both the early/intermediate and advanced stages of AMD.

Notably, this study revealed that the HBV carrier status increased the risk of early/intermediate AMD. Several studies have reported an association between HBV infection and AMD in Korean and Taiwanese populations [16,17,33]. Viral hepatitis can lead to systemic inflammation and oxidative stress [34,35], which are factors that might increase the AMD risk [36]. Additionally, HBsAg shares homology with retinal S antigen and induces experimental autoimmune uveitis in rats [37], potentially contributing to AMD development. Given that the prevalence rates of HBV carriers in Korea and Taiwan are greater than those in major Western countries [38], the impact of viral hepatitis on the development of AMD in the Korean and Taiwanese populations is likely to be more substantial.

This study is the first to identify a negative association between the serum albumin concentration and the risk of advanced AMD. More than 70% of the free radical-trapping activity in serum is attributable to the multiple ligand-binding capacities and free radical-trapping properties of albumin [39]. Oxidative stress and oxidative damage are associated with the development of age-related diseases [40], which explains our finding that increased serum albumin levels are associated with a decreased risk of advanced AMD.

Moreover, for the first time, we observed a correlation between higher serum inorganic phosphate levels and an increased risk of advanced AMD. Hyperphosphatemia can result from kidney dysfunction, excessive intake of phosphate-containing substances, hormonal disorders like hypoparathyroidism, or conditions causing rapid cell breakdown, such as tumor lysis syndrome [41]. Additionally, certain drugs, such as penicillin, steroids, loop diuretics, and thiazides, can contribute to the development of hyperphosphatemia as a side effect [42]. Even within the normal range, higher serum inorganic phosphate levels have been linked to the development of vascular disease [43]. This process may occur through mechanisms that induce vascular calcification in larger and medium-sized vessels and initiate endothelial dysfunction, potentially underlying the association between AMD and serum inorganic phosphate levels. Notably, zinc supplementation has been reported to inhibit phosphate-induced vascular calcification [44], which may be a possible mechanism for preventing the progression to advanced AMD, as noted in the AREDS studies [45]. The previously discussed conditions associated with hyperphosphatemia warrant investigation to elucidate their potential role in increasing the risk of AMD. Moreover, further research is necessary to evaluate whether interventions such as dietary phosphate restriction, the use of phosphate binders, or dialysis in patients with kidney failure—aimed at reducing serum inorganic phosphate levels—could contribute to mitigating the risk of AMD.

The following associations identified in our research, though noteworthy, exhibited an OR proximal to 1, necessitating further investigation to substantiate their clinical implications. A higher MCV was associated with a risk of early/intermediate AMD. An elevated MCV, referred to as macrocytosis, has been linked to various factors, including deficiencies in vitamin B12 and folic acid [46], which could increase the risk of AMD [47]. Higher serum UA levels reduce the risk of early/intermediate AMD, in accordance with the increasing body of evidence supporting the antioxidant and anti-inflammatory properties of UA [48]. These properties likely contribute to the protective effect of serum UA against AMD.

Interestingly, in this study, a significant association between the smoking status and AMD was not detected. The tendency for the association between AMD and smoking status to be weaker in the Korean population than in other studies has also been noted in previous Korean population-based studies using KNHANES data [2,16,17]. In our study, the smoking status was strongly associated with both early/intermediate and advanced AMD in the univariable analysis; however, this association disappeared in the multivariable model. This difference may be attributed to the possibility of collinearity between the smoking status and other variables. The smoking status differs significantly between sexes in the Korean population [49], which is a unique characteristic compared with other populations. Considering this information, the strong collinearity between sex and the smoking status in Koreans could have contributed to the loss of statistical significance for the smoking status when both variables were included in the multivariable model.

Our study has several limitations that warrant consideration. First, while the rate of missing data are within acceptable limits, it has the potential to affect the reliability of our findings. We addressed this concern by employing multiple imputation techniques to address missing data and provided both original (non-imputed) and imputed data to ensure the robustness of our results. Second, health screening center-based studies may exhibit participation-related bias, as these individuals might be more health conscious or belong to specific socioeconomic groups [50]. Acknowledging this limitation, we implemented statistical weights to adjust for the skewed representation of the general population within our data, thereby ensuring that our findings more accurately reflect the composition of the general population [15]. Finally, diagnosing AMD based on fundus findings in individuals as young as their 30s in this study is somewhat controversial. While most studies diagnose AMD starting in the 40s or 50s, there is no established age criterion for an AMD diagnosis. Furthermore, some studies have included participants in their 30s [9,51], similar to our research. The observed prevalence of 3.34% among individuals in their 30s in our study is noteworthy and valuable to report. Future studies should conduct further analyses to better understand the characteristics of AMD in this younger age group.

## 5. Conclusions

The weighted prevalence of AMD among Koreans aged 30 years and older is 15.42%, whereas that among those in their 30s is 3.34%. In addition to age, male sex, HTN, MCV, HBV carrier status, and serum UA levels, which are known to be associated with AMD, this study has, for the first time, established associations of AMD with retinal arteriosclerosis, serum inorganic phosphate levels, and total albumin levels. Additional studies are needed to validate these findings in non-Korean populations.

## Figures and Tables

**Table 1:** Information on subjects’ demographics, social history, medical history, laboratory data, and retinal arteriolar sclerosis before multiple imputation.

Variables	Total Study Population (n = 73,574)	No Age-Related Macular Degeneration (n = 64,692)	Overall Age-Related Macular Degeneration (n = 8882)	*p* Value ^1^
Age group, no. (%)				
30–40 years	15,830 (21.52%)	15,227 (23.54%)	603 (6.79%)	<0.001
40–49 years	25,331 (34.43%)	23,073 (35.67%)	2258 (25.42%)	
50–59 years	20,967 (28.50%)	17,804 (27.52%)	3163 (35.61%)	
60–69 years	9424 (12.81%)	7243 (11.20%)	2181 (24.56%)	
≥70 years	2022 (2.75%)	1345 (2.08%)	677 (7.62%)	
Sex, no. (%)				
Male	39,308 (53.43%)	33,627 (51.98%)	5681 (63.96%)	<0.001
Female	34,266 (46.57%)	31,065 (48.02%)	3201 (36.04%)	
Smoking, no. (%)				
Never	37,880 (53.35%)	33,829 (54.19%)	4051 (47.24%)	<0.001
Former	17,603 (24.79%)	14,998 (24.03%)	2605 (30.38%)	
Current	15,517 (21.86%)	13,597 (21.78%)	1920 (22.39%)	
Drinking, no. (%)				
Never	20,395 (28.90%)	17,980 (28.94%)	2415 (28.58%)	0.586
Former	2192 (3.11%)	1917 (3.09%)	275 (3.25%)	
Current	47,984 (67.99%)	42,223 (67.97%)	5761 (68.17%)	
Active lifestyle, no. (%)	47,433 (65.57%)	41,289 (64.90%)	6144 (70.40%)	<0.001
DM, no. (%)	7360 (10.00%)	6048 (9.35%)	1312 (14.77%)	<0.001
HTN, no. (%)	19,735 (26.83%)	16,366 (25.30%)	3369 (37.94%)	<0.001
Dyslipidemia, no. (%)	28,208 (38.34%)	24,498 (37.87%)	3710 (41.78%)	<0.001
Cardiovascular diseases, no. (%)	2585 (3.52%)	2143 (3.32%)	442 (4.98%)	<0.001
Cerebrovascular diseases, no. (%)	342 (0.47%)	278 (0.43%)	64 (0.72%)	<0.001
Cancer, no. (%)	1933 (2.63%)	1647 (2.55%)	286 (3.22%)	<0.001
Income, no. (%)				
<KRW 3,000,000	10,785 (15.98%)	9103 (15.32%)	1682 (20.90%)	<0.001
KRW 3,000,000–5,000,000	14,735 (21.84%)	13,088 (22.02%)	1647 (20.46%)	
KRW 5,000,000–10,000,000	23,854 (35.35%)	21,225 (35.72%)	2629 (32.67%)	
≥KRW 10,000,000	18,101 (26.83%)	16,011 (26.94%)	2090 (25.97%)	
Education, no. (%)				
<Middle school graduate	9199 (12.76%)	7659 (12.07%)	1540 (17.76%)	<0.001
Middle school graduate	13,383 (18.56%)	11,517 (18.16%)	1866 (21.52%)	
High school graduate	33,653 (46.68%)	30,055 (47.38%)	3598 (41.50%)	
≥College/university graduate	15,865 (22.20%)	14,199 (22.39%)	1666 (19.22%)	
Height (cm)				
Female	157.96 ± 5.38	158.12 ± 5.35	156.41 ± 5.44	<0.001
Male	170.31 ± 5.74	170.49 ± 5.71	169.2 ± 5.78	<0.001
BMI (kg/m^2^)				
Female	22.36 ± 2.92	22.29 ± 2.91	23.02 ± 2.97	<0.001
Male	24.52 ± 2.75	24.53 ± 2.76	24.43 ± 2.67	0.009
Blood laboratory parameters				
Corrected calcium (mg/dL)	9.48 ± 0.49	9.48 ± 0.49	9.48 ± 0.48	0.219
P (mg/dL)	3.66 ± 0.58	3.66 ± 0.58	3.64 ± 0.62	0.062
BUN (mg/dL)	13.67 ± 3.64	13.56 ± 3.61	14.41 ± 3.71	<0.001
Cr (mg/dL)	1.00 ± 0.20	1.00 ± 0.21	1.03 ± 0.19	<0.001
AST (U/L)	24.43 ± 15.63	24.21 ± 15.68	26.05 ± 15.20	<0.001
ALT (U/L)	26.10 ± 24.89	25.90 ± 25.10	27.59 ± 23.27	<0.001
ALP (U/L)	62.10 ± 19.91	61.69 ± 19.85	65.14 ± 20.08	<0.001
GGT (U/L)	34.73 ± 44.33	34.14 ± 43.27	39.02 ± 51.22	<0.001
Total bilirubin (mg/dL)	1.07 ± 0.45	1.07 ± 0.45	1.09 ± 0.44	0.001
UA (mg/dL)	5.55 ± 1.44	5.53 ± 1.43	5.69 ± 1.46	<0.001
Total cholesterol (mg/dL)	195.60 ± 34.49	195.38 ± 34.47	197.18 ± 34.56	<0.001
HDL (mg/dL)	54.21 ± 13.41	54.35 ± 13.43	53.16 ± 13.22	<0.001
TG (mg/dL)	115.12 ± 78.35	114.51 ± 77.27	119.63 ± 85.66	<0.001
Total albumin (g/dL)	4.38 ± 0.26	4.38 ± 0.26	4.35 ± 0.27	<0.001
Hs-CRP (mg/dL)	0.14 ± 0.46	0.14 ± 0.43	0.17 ± 0.61	<0.001
Hb (g/dL)	14.33 ± 1.62	14.31 ± 1.63	14.53 ± 1.55	<0.001
WBC (×10^3^/μL)	5.75 ± 1.68	5.75 ± 1.68	5.79 ± 1.67	0.017
MCV (fL)	92.35 ± 4.90	92.24 ± 4.90	93.17 ± 4.76	<0.001
Platelet (×10^3^/μL)	242.27 ± 55.05	243.08 ± 54.93	236.32 ± 55.55	<0.001
HbA1c (%)	5.75 ± 0.69	5.73 ± 0.68	5.86 ± 0.76	<0.001
HBsAg-positive, no. (%)	3326 (4.53%)	2787 (4.31%)	539 (6.08%)	<0.001
Anti-HCV Ab-positive, no. (%)	818 (1.11%)	657 (1.02%)	161 (1.82%)	<0.001
*H. pylori* Ab-positive, no. (%)	36,945 (56.95%)	32,392 (56.85%)	4553 (57.71%)	0.149
Retinal arteriolar sclerosis, no. (%)				
Grade 0	60,085 (81.99%)	53,941 (83.38%)	6361 (71.62%)	<0.001
Grade 1	10,554 (14.40%)	8661 (13.39%)	1956 (22.02%)	
Grade 2	2338 (3.19%)	1840 (2.84%)	504 (5.67%)	
Grade 3	296 (0.40%)	239 (0.37%)	58 (0.65%)	
Grade 4	14 (0.02%)	11 (0.02%)	3 (0.03%)	

Ab = antibody; ALP = alkaline phosphatase; ALT = alanine transaminase; AST = aspartate transaminase; BMI = body mass index; BUN = blood urea nitrogen; Cr = creatinine; DM = diabetes mellitus; GGT = gamma-glutamyl transferase; HBsAg = hepatitis B surface antigen; HCV = hepatitis C; Hb = hemoglobin; HbA1c = glycosylated hemoglobin; HDL = high-density lipoprotein; *H. pylori* = *Helicobacter pylori*; Hs-CRP = high-sensitivity C-reactive protein; HTN = hypertension; KRW = South Korean won; MCV = mean corpuscular volume; P = inorganic phosphate; TG = triglyceride; UA = uric acid; WBC = white blood cell. ^1^ No age-related macular degeneration vs. overall age-related macular degeneration.

**Table 2:** Weighted prevalence of age-related macular degeneration in the Gangnam Eye Cohort (2003–2010).

	Total, Weighted % (95% CI)	Age Group (Years)					
		30–39 Years, Weighted % (95% CI)	40–49 Years, Weighted % (95% CI)	50–59 Years, Weighted % (95% CI)	60–69 Years, Weighted % (95% CI)	≥70 Years, Weighted % (95% CI)	*p* Value ^1^
Early Age-Related Macular Degeneration	13.72 (13.47–13.97)	3.21 (2.93–3.48)	8.37 (7.97–8.77)	14.42 (13.91–14.94)	20.86 (20.12–21.61	30.92 (30.02–31.83)	<0.001
Intermediate Age-Related Macular Degeneration	1.31 (1.23–1.39)	0.09 (0.04–0.14)	0.23 (0.16–0.30)	0.67 (0.55–0.79)	1.75 (1.51–1.99)	5.87 (5.41–6.33)	<0.001
Advanced Age-Related Macular Degeneration	0.39 (0.35–0.44)	0.04 (0.01–0.07)	0.09 (0.05–0.14)	0.15 (0.10–0.21)	0.60 (0.46–0.75)	1.69 (1.43–1.94)	<0.001
Overall Age-Related Macular Degeneration	15.42 (15.16–15.68)	3.34 (3.06–3.61)	8.70 (8.29–9.11)	15.25 (14.72–15.78)	23.22 (22.44–23.99)	38.47 (37.52–39.43)	<0.001

CI = confidence interval. ^1^ Cochran–Armitage trend test for age groups

**Table 3:** Logistic regression analyses for associations between and early/intermediate age-related macular degeneration.

	Early/Intermediate Age-Related Macular Degeneration				
Variables	Univariable Analysis ^1^		Multivariable Model ^1,2^		
	Odds Ratio (95% Confidence Interval)	*p* Value	Odds Ratio (95% Confidence Interval)	Original *p* Value	Benjamini–Hochberg-Corrected *p* Value
Age group, no. (%)					
30–39 years	1 (reference)		1 (reference)		
40–49 years	2.47 (2.25–2.70)	<0.001	2.30 (2.09–2.53)	<0.001	<0.001
50–59 years	4.48 (4.10–4.90)	<0.001	3.86 (3.50–4.26)	<0.001	<0.001
60–69 years	7.46 (6.79–8.21)	<0.001	5.89 (5.29–6.56)	<0.001	<0.001
≥70 years	12.25 (10.82–13.87)	<0.001	9.32 (8.10–10.72)	<0.001	<0.001
Sex, no. (%)					
Female	1 (reference)		1 (reference)		
Male	1.63 (1.56–1.71)	<0.001	1.83 (1.67–2.00)	<0.001	<0.001
Smoking, no. (%)					
Never	1 (reference)		1 (reference)		
Former	1.42 (1.35–1.50)	<0.001	0.98 (0.91–1.06)	0.592	0.744
Current	1.17 (1.11–1.24)	<0.001	1.06 (0.98–1.15)	0.142	0.345
Drinking, no. (%)					
Never	1 (reference)				
Former	1.06 (0.92–1.21)	0.430			
Current	1.02 (0.97–1.07)	0.521			
Active lifestyle, no. (%)	1.28 (1.22–1.34)	<0.001	1.00 (0.95–1.05)	0.969	0.990
DM, no. (%)	1.67 (1.57–1.78)	<0.001	1.01 (0.93–1.11)	0.760	0.880
HTN, no. (%)	1.79 (1.71–1.87)	<0.001	1.12 (1.06–1.18)	<0.001	<0.001
Dyslipidemia, no. (%)	1.18 (1.13–1.23)	<0.001	0.96 (0.91–1.01)	0.149	0.345
Cardiovascular diseases, no. (%)	1.51 (1.36–1.68)	<0.001	0.97 (0.87–1.09)	0.645	0.767
Cerebrovascular diseases, no. (%)	1.66 (1.26–2.18)	<0.001	0.92 (0.69–1.23)	0.576	0.744
Cancer, no. (%)	1.27 (1.12–1.44)	<0.001	1.10 (0.96–1.25)	0.182	0.364
Income, no. (%)					
<KRW 3,000,000	1 (reference)		1 (reference)		
KRW 3,000,000–5,000,000	0.71 (0.66–0.76)	<0.001	0.96 (0.88–1.04)	0.314	0.531
KRW 5,000,000–10,000,000	0.70 (0.65–0.75)	<0.001	0.95 (0.87–1.03)	0.176	0.364
≥KRW 10,000,000	0.74 (0.69–0.79)	<0.001	0.94 (0.86–1.02)	0.118	0.305
Education level, no. (%)					
<Middle school graduate	1 (reference)		1 (reference)		
Middle school graduate	0.81 (0.75–0.87)	<0.001	1.00 (0.92–1.09)	0.990	0.990
High school graduate	0.61 (0.57–0.65)	<0.001	0.87 (0.80–0.94)	0.001	0.004
≥College/university graduate	0.59 (0.55–0.64)	<0.001	0.80 (0.73–0.88)	<0.001	<0.001
Height (cm)	1.00 (1.00–1.00)	0.745			
BMI (kg/m^2^)	1.05 (1.04–1.06)	<0.001	1.00 (1.00–1.01)	0.413	0.606
Blood laboratory parameters					
Corrected calcium (mg/dL)	0.98 (0.93–1.02)	0.303			
P (mg/dL)	0.96 (0.92–1.00)	0.033	1.02 (0.98–1.06)	0.341	0.536
BUN mg/dl (mg/dL)	1.06 (1.05–1.07)	<0.001	1.01 (1.00–1.01)	0.072	0.198
Cr (mg/dL)	2.00 (1.80–2.22)	<0.001	0.86 (0.74–1.01)	0.060	0.176
AST (U/L)	1.01 (1.00–1.01)	<0.001	1.00 (1.00–1.00)	0.268	0.472
ALT (U/L)	1.00 (1.00–1.00)	<0.001	1.00 (1.00–1.00)	0.586	0.744
ALP (U/L)	1.01 (1.01–1.01)	<0.001	1.00 (1.00–1.00)	0.224	0.411
GGT (U/L)	1.00 (1.00–1.00)	<0.001	1.00 (1.00–1.00)	0.904	0.990
Total bilirubin (mg/dL)	1.09 (1.04–1.14)	0.001	1.02 (0.97–1.08)	0.485	0.688
UA (mg/dL)	1.07 (1.06–1.09)	<0.001	0.97 (0.95–0.99)	0.004	0.014
Total cholesterol (mg/dL)	1.00 (1.00–1.00)	<0.001	1.00 (1.00–1.00)	0.966	0.990
HDL (mg/dL)	0.99 (0.99–1.00)	<0.001	1.00 (1.00–1.00)	0.182	0.364
TG (mg/dL)	1.00 (1.00–1.00)	<0.001	1.00 (1.00–1.00)	0.205	0.392
Total albumin (g/dL)	0.66 (0.61–0.72)	<0.001	0.97 (0.88–1.07)	0.520	0.715
Hs-CRP (mg/dL)	1.06 (1.02–1.11)	0.007	1.01 (0.97–1.06)	0.632	0.767
Hb (g/dL)	1.09 (1.08–1.11)	<0.001	1.00 (0.98–1.02)	0.975	0.990
WBC (×10^3^/μL)	1.02 (1.00–1.03)	0.019	0.99 (0.98–1.01)	0.343	0.536
MCV (fL)	1.04 (1.04–1.05)	<0.001	1.01 (1.01–1.02)	<0.001	0.001
Platelet (×10^3^/μL)	1.00 (1.00–1.00)	<0.001	1.00 (1.00–1.00)	0.353	0.536
HbA1c (%)	1.23 (1.20–1.26)	<0.001	1.00 (0.96–1.05)	0.865	0.976
HBsAg-positive	1.45 (1.32–1.60)	<0.001	1.47 (1.33–1.63)	<0.001	<0.001
Anti-HCV Ab-positive	1.78 (1.50–2.12)	<0.001	1.23 (1.03–1.48)	0.025	0.079
*H. pylori* Ab-positive	1.04 (0.99–1.09)	0.106			
Retinal arteriolar sclerosis (n, %)					
No	1 (reference)		1 (reference)		
Low	1.91 (1.81–2.02)	<0.001	1.35 (1.28–1.42)	<0.001	<0.001
High	2.21 (2.01–2.44)	<0.001	1.34 (1.21–1.49)	<0.001	<0.001

Ab = antibody; ALP = alkaline phosphatase; ALT = alanine transaminase; AST = aspartate transaminase; BMI = body mass index; BUN = blood urea nitrogen; Cr = creatinine; DM = diabetes mellitus; GGT = gamma-glutamyl transferase; HBsAg = hepatitis B surface antigen; HCV = hepatitis C; Hb = hemoglobin; HbA1c = glycosylated hemoglobin; HDL = high-density lipoprotein; *H. pylori* = *Helicobacter pylori*; Hs-CRP = high-sensitivity C-reactive protein; HTN = hypertension; KRW = South Korean won; MCV = mean corpuscular volume; P = inorganic phosphate; TG = triglyceride; UA = uric acid; WBC = white blood cell. ^1^ Results are based on imputed data. ^2^ Adjusted for age; sex; smoking status; physical activity level; a history of diabetes, hypertension, dyslipidemia, heart disease, stroke, or cancer; monthly household income; education level; body mass index; serum levels of inorganic phosphate, BUN, creatinine, AST, ALT, ALP, GGT, total bilirubin, uric acid, total cholesterol, HDL, TG, total albumin, Hs-CRP, hemoglobin, and HbA1c; WBC counts; MCV; platelet counts; HBsAg; anti-HCV; and the retinal arteriosclerosis grade.

**Table 4:** Logistic regression analyses of associations between potential risk factors and advanced age-related macular degeneration.

	Advanced Age-Related Macular Degeneration				
Variables	Univariable Analysis ^1^		Multivariable Model ^1,2^		
	Odds Ratio (95% Confidence Interval)	*p* Value	Odds Ratio (95% Confidence Interval)	Original *p* Value	Benjamini–Hochberg–Corrected *p* Value
Age group, no. (%)					
30–39 years	1 (reference)		1 (reference)		
40–49 years	3.17 (1.21–8.30)	0.019	2.49 (0.94–6.59)	0.067	0.195
50–59 years	5.13 (1.99–13.23)	0.001	3.14 (1.18–8.35)	0.022	0.092
60–69 years	24.39 (9.78–60.82)	<0.001	11.88 (4.49–31.44)	<0.001	<0.001
≥70 years	67.93 (26.31–175.36)	<0.001	29.21 (10.44–81.68)	<0.001	<0.001
Sex, no. (%)					
Female	1 (reference)		1 (reference)		
Male	2.31 (1.61–3.30)	<0.001	2.88 (1.73–4.79)	<0.001	<0.001
Smoking, no. (%)					
Never	1 (reference)		1 (reference)		
Former	2.06 (1.44–2.97)	<0.001	1.14 (0.74–1.77)	0.550	0.765
Current	1.19 (0.77–1.86)	0.437	1.10 (0.66–1.82)	0.721	0.876
Drinking, no. (%)					
Never	1 (reference)				
Former	1.55 (0.69–3.46)	0.289			
Current	0.96 (0.67–1.38)	0.822			
Active lifestyle, no. (%)	1.46 (1.01–2.11)	0.045	1.05 (0.71–1.55)	0.802	0.904
DM, no. (%)	2.18 (1.76–2.70)	<0.001	0.74 (0.41–1.32)	0.304	0.59
HTN, no. (%)	3.25 (2.75–3.83)	<0.001	1.51 (1.05–2.16)	0.026	0.092
Dyslipidemia, no. (%)	1.13 (0.95–1.35)	0.686			
Cardiovascular diseases, no. (%)	2.59 (1.92–3.51)	0.002	1.11 (0.61–2.05)	0.730	0.876
Cerebrovascular diseases, no. (%)	3.18 (1.56–6.50)	0.105			
Cancer, no. (%)	1.63 (0.72–3.69)	0.243			
Income, no. (%)					
<KRW 3,000,000	1 (reference)		1 (reference)		
KRW 3,000,000–5,000,000	0.46 (0.28–0.76)	0.002	0.76 (0.44–1.30)	0.311	0.592
KRW 5,000,000–10,000,000	0.50 (0.33–0.77)	0.002	0.94 (0.58–1.55)	0.819	0.904
≥KRW 10,000,000	0.45 (0.28–0.73)	0.001	0.81 (0.47–1.40)	0.454	0.692
Education level, no. (%)					
<Middle school graduate	1 (reference)		1 (reference)		
Middle school graduate	0.69 (0.43–1.11)	0.125	1.00 (0.60–1.67)	0.993	0.993
High school graduate	0.38 (0.25–0.60)	<0.001	0.73 (0.44–1.23)	0.235	0.537
≥College/university graduate	0.47 (0.29–0.78)	0.004	0.82 (0.46–1.48)	0.515	0.749
Height (cm)	1.00 (0.98–1.02)	0.965			
BMI (kg/m^2^)	1.05 (1.00–1.11)	0.057	0.97 (0.92–1.04)	0.400	0.674
Blood laboratory parameters					
Corrected calcium (mg/dL)	0.74 (0.62–0.88)	0.077	1.25 (0.81–1.92)	0.320	0.592
P (mg/dL)	1.30 (1.16–1.47)	0.028	1.45 (1.18–1.77)	<0.001	0.002
BUN mg/dl (mg/dL)	1.08 (1.07–1.09)	<0.001	1.05 (1.01–1.09)	0.025	0.092
Cr (mg/dL)	1.72 (1.49–1.97)	<0.001	0.56 (0.26–1.21)	0.140	0.373
AST (U/L)	1.00 (1.00–1.01)	0.793			
ALT (U/L)	1.00 (1.00–1.00)	0.842			
ALP (U/L)	1.00 (1.00–1.01)	0.019	1.00 (0.99–1.00)	0.179	0.441
GGT (U/L)	1.00 (1.00–1.00)	<0.001	1.00 (1.00–1.00)	0.038	0.122
Total bilirubin (mg/dL)	0.94 (0.78–1.14)	0.760			
UA (mg/dL)	1.12 (1.09–1.16)	0.001	1.01 (0.88–1.16)	0.900	0.960
Total cholesterol (mg/dL)	1.00 (1.00–1.00)	0.633			
HDL (mg/dL)	1.00 (0.99–1.01)	0.942			
TG (mg/dL)	1.00 (1.00–1.00)	0.403			
Total albumin (g/dL)	0.18 (0.13–0.24)	<0.001	0.30 (0.15–0.60)	0.001	0.005
Hs-CRP (mg/dL)	1.18 (0.96–1.46)	0.117			
Hb (g/dL)	0.96 (0.91–1.00)	0.362			
WBC (×10^3^/μL)	1.02 (0.97–1.06)	0.720			
MCV (fL)	1.04 (1.02–1.05)	0.012	0.99 (0.95–1.02)	0.434	0.692
Platelet (×10^3^/μL)	1.00 (1.00–1.00)	0.075	1.00 (1.00–1.00)	0.959	0.990
HbA1c (%)	1.34 (1.17–1.53)	<0.001	1.04 (0.81–1.35)	0.739	0.876
HBsAg-positive	0.46 (0.26–0.83)	0.187			
Anti-HCV Ab-positive	2.72 (1.64–4.53)	0.049	1.23 (0.45–3.42)	0.688	0.876
*H. pylori* Ab-positive	0.80 (0.57–1.11)	0.184			
Retinal arteriolar sclerosis (n, %)					
No	1 (reference)		1 (reference)		
Low	2.36 (1.58–3.53)	<0.001	1.23 (0.82–1.84)	0.333	0.592
High	8.01 (5.19–12.36)	<0.001	3.14 (1.97–5.01)	<0.001	<0.001

Ab = antibody; ALP = alkaline phosphatase; ALT = alanine transaminase; AST = aspartate transaminase; BMI = body mass index; BUN = blood urea nitrogen; Cr = creatinine; DM = diabetes mellitus; GGT = gamma-glutamyl transferase; HBsAg = hepatitis B surface antigen; HCV = hepatitis C; Hb = hemoglobin; HbA1c = glycosylated hemoglobin; HDL = high-density lipoprotein; *H. pylori* = *Helicobacter pylori*; Hs-CRP = high-sensitivity C-reactive protein; HTN = hypertension; KRW = South Korean won; MCV = mean corpuscular volume; P = inorganic phosphate; TG = triglyceride; UA = uric acid; WBC = white blood cell. ^1^ Results are based on imputed data. ^2^ Adjusted for age; sex; smoking status; physical activity level; a history of diabetes, hypertension, or heart disease; monthly household income; education level; body mass index; serum levels of corrected calcium, inorganic phosphate, BUN, creatinine, ALP, GGT, uric acid, total albumin, and HbA1c; MCV; platelet counts; anti-HCV; and the retinal arteriosclerosis grade.

## Data Availability

The original contributions presented in the study are included in the article and Supplementary Materials, and further inquiries can be directed to the corresponding author.

## Supplementary Materials

The following supporting information can be downloaded at https://www.mdpi.com/article/10.3390/biomedicines12122681/s1: Table S1: Distribution of missingness by variable; Table S2: Information on subjects’ demographics, social history, medical history, laboratory data, and retinal arteriolar sclerosis after multiple imputation (pooled); Table S3: Characteristics of normal participants and participants with early/intermediate age-related macular degeneration after multiple imputation and associations with potential risk factors identified via logistic regression analysis before multiple imputation; Table S4: Characteristics of normal participants and participants with advanced age-related macular degeneration after multiple imputation and associations with potential risk factors identified via logistic regression analysis before multiple imputation. Table S5: Characteristics of Normal and Advanced Age-Related Macular Degeneration Participants before and after Multiple Imputation. Table S6: Logistic Regression Analyses for Associations between and Advanced Age-Related Macular Degeneration before Multiple Imputation.
