# Supplementary material for: A Comprehensive Health Screening Program Reveals the Prevalence of and Risk Factors for Age-Related Macular Degeneration: A Cross-Sectional Analysis
Source: Biomedicines. 2024 Nov 25;12(12):2681. doi: 10.3390/biomedicines12122681 (PMC11727633; doi:10.3390/biomedicines12122681)
Supplement: Supplementary file 1 [file biomedicines-12-02681-s001.zip › biomedicines-3280443-supplementary.pdf]

Table S1. Distribution of missingness by variable.

Variables	Missing records, no. (%)
Age Group	0 (0.00%)
Gender	0 (0.00%)
Smoking	2574 (3.50%)
Drinking	3003 (4.08%)
Physical activity level	1231 (1.67%)
DM	7 (0.01%)
HTN	9 (0.01%)
Dyslipidemia	9 (0.01%)
Cardiovascular diseases	99 (0.13%)
Cerebrovascular diseases	316 (0.43%)
Cancer	0 (0.00%)
Income	6099 (8.29%)
Education	1474 (2.00%)
Height	244 (0.33%)
BMI	245 (0.33%)
Blood laboratory parameters	
Corrected calcium (mg/dL)	59 (0.08%)
P (mg/dL)	54 (0.07%)
BUN (mg/dL)	51 (0.07%)
Cr (mg/dL)	54 (0.07%)
AST (U/L)	54 (0.07%)
ALT (U/L)	55 (0.07%)
ALP (U/L)	57 (0.08%)
GGT (U/L)	118 (0.16%)
Total bilirubin (mg/dL)	55 (0.07%)
UA (mg/dL)	57 (0.08%)
Total cholesterol (mg/dL)	48 (0.07%)
HDL (mg/dL)	76 (0.10%)
TG (mg/dL)	77 (0.10%)
Total albumin (g/dL)	55 (0.07%)
Hs-CRP (mg/dL)	12,477 (16.96%)
Hb (g/dL)	308 (0.42%)
WBC (×10^3/μL)	57 (0.08%)
MCV (fL)	57 (0.08%)
Platelet (×10^3/μL)	307 (0.42%)
HbA1c (%)	2934 (3.99%)
HBsAg-positive, no. (%)	110 (0.15%)
Anti-HCV Ab-positive, no. (%)	104 (0.14%)
H. pylori Ab-positive, no. (%)	8702 (11.83%)
Keith-Wagener-Barker classification of Retinal Arteriolar Sclerosis	0 (0.00%)

Ab = antibody; ALP = alkaline phosphatase ; ALT = alanine transaminase; AST = aspartate transaminase; BMI = body mass index; BUN = blood urea nitrogen; Cr = creatinine; DM = diabetes mellitus; GGT = gamma-glutamyl transferase; HBsAg = hepatitis B surface antigen; HCV = hepatitis C; Hb = hemoglobin; HbA1c = glycosylated hemoglobin; HDL = high-density lipoprotein; *H.pylori* = *Helicobacter pylori*; Hs-CRP = high-sensitivity c-reactive protein; HTN = hypertension; KRW = South Korean won; MCV = mean corpuscular volume ; P = inorganic phosphate; TG = triglyceride; UA = uric acid; WBC = white blood count.

Table S2. Subject information on Demographics, Social History, Medical History, Laboratory Data, and Retinal Arteriolar Sclerosis After Multiple Imputation (Pooled).

Variables	Total Study Population (n = 73,574)	No Age-Related Macular Degeneration (n = 64,692)	Overall Age-Related Macular Degeneration (n = 8882)	P Value[1]
Age group, no. (%)				
30-40 yrs	15,830.00 (21.52%)	152,27.00 (23.54%)	603.00 (6.79%)	
40-49 yrs	25,331.00 (34.43%)	23,073.00 (35.67%)	2258.00 (25.42%)	
50-59 yrs	20,967.00 (28.50%)	17,804.00 (27.52%)	3163.00 (35.61%)	<0.001
60-69 yrs	9424.00 (12.81%)	7243.00 (11.20%)	2181.00 (24.56%)	
≥70 yrs	2022.00 (2.75%)	1345.00 (2.08%)	677.00 (7.62%)	
Sex, no. (%)				
Male	39,308 (53.43%)	33,627 (51.98%)	5681 (63.96%)	<0.001
Female	34,266 (46.57%)	31,065 (48.02%)	3201 (36.04%)	
Smoking, no. (%)				
Never	39,275.90 (53.38%)	35,059.00 (54.19%)	4216.90 (47.48%)	
Former	18,231.10 (24.78%)	15,549.90 (24.04%)	2681.20 (30.19%)	<0.001
Current	16,067.10 (21.84%)	14,083.20 (21.77%)	1983.90 (22.34%)	
Drinking, no. (%)				
Never	21,277.20 (28.92%)	18,737.20 (28.96%)	2540.10 (28.60%)	
Former	2284.80 (3.11%)	1996.70 (3.09%)	288.10 (3.24%)	0.468
Current	50,012.10 (67.98%)	43,958.20 (67.95%)	6053.90 (68.16%)	
Active lifestyle, no. (%)	48,241.70 (65.57%)	4,1996.20 (64.92%)	6245.50 (70.32%)	<0.001
DM, no. (%)	7360.70 (10.00%)	6048.60 (9.35%)	1312.10 (14.77%)	<0.001
HTN, no. (%)	19,737.50 (26.83%)	16,367.90 (25.30%)	3369.60 (37.94%)	<0.001
Dyslipidemia, no. (%)	28,211.10 (38.34%)	24,500.60 (37.87%)	3710.50 (41.78%)	<0.001
Cardiovascular diseases, no. (%)	2588.00 (3.52%)	2145.80 (3.32%)	442.20 (4.98%)	<0.001
Cerebrovascular diseases, no. (%)	343.70 (0.47%)	279.40 (0.43%)	64.30 (0.72%)	<0.001
Cancer, no. (%)	1933.00 (2.63%)	1647.00 (2.55%)	286.00 (3.22%)	<0.001
Income, no. (%)				
<KRW 3,000,000	11,773.50 (16.00%)	9957.30 (15.39%)	1816.20 (20.45%)	
KRW 3,000,000-5,000,000	16,072.90 (21.85%)	14,242.90 (22.02%)	1830.00 (20.60%)	
KRW 5,000,000-10,000,000	26,004.40 (35.34%)	23,081.80 (35.68%)	2922.60 (32.90%)	<0.001
≥KRW 10,000,000	19,723.40 (26.81%)	17,410.00 (26.91%)	2313.40 (26.05%)	
Education, no. (%)				
<Middle school graduate	9384.90 (12.76%)	7817.60 (12.08%)	1567.30 (17.65%)	
Middle school graduate	13,652.30 (18.56%)	11,748.00 (18.16%)	1904.40 (21.44%)	<0.001
High school graduate	34,347.30 (46.68%)	30,650.10 (47.38%)	3697.30 (41.63%)	

≥College/university graduate	16,189.60 (22.00%)	14,476.50 (22.38%)	1713.10 (19.29%)	
Height (cm)				
Female	157.98±5.41	158.14±5.37	156.43±5.48	<0.001
Male	170.29±5.75	170.48±5.72	169.18±5.80	<0.001
BMI (kg/m^2)				
Female	22.36±2.93	22.30±2.91	23.02±2.97	<0.001
Male	24.52±2.75	24.53±2.76	24.43±2.67	<0.001
Blood laboratory parameters				
Corrected calcium (mg/dL)	9.48±0.49	9.36±0.46	9.59±0.49	0.217
P (mg/dL)	3.66±0.58	3.80±0.58	3.53±0.55	0.063
BUN (mg/dL)	13.67±3.64	12.95±3.63	14.29±3.52	<0.001
Cr (mg/dL)	1.00±0.20	0.87±0.15	1.12±0.17	<0.001
AST (U/L)	24.43±15.63	22.16±15.14	26.40±15.77	<0.001
ALT (U/L)	26.10±24.89	20.08±21.77	31.34±26.21	<0.001
ALP (U/L)	62.10±19.91	59.48±20.30	64.39±19.27	<0.001
GGT (U/L)	34.75±44.32	20.75±24.33	46.95±53.29	<0.001
Total bilirubin (mg/dL)	1.07±0.45	0.93±0.36	1.20±0.48	0.001
UA (mg/dL)	5.55±1.44	4.71±1.19	6.28±1.22	<0.001
Total cholesterol (mg/dL)	195.60±34.49	194.56±34.74	196.50±34.24	<0.001
HDL (mg/dL)	54.21±13.41	59.42±13.61	49.67±11.44	<0.001
TG (mg/dL)	115.14±78.33	91.45±54.18	135.78±89.51	<0.001
Total albumin (g/dL)	4.38±0.26	4.31±0.24	4.43±0.27	<0.001
Hs-CRP (mg/dL)	0.19±0.45	0.16±0.36	0.21±0.51	0.004
Hb (g/dL)	14.33±1.62	13.08±1.14	15.43±1.10	<0.001
WBC (×10^3/μL)	5.75±1.68	5.37±1.49	6.09±1.77	0.018
MCV (fL)	92.35±4.90	91.71±5.30	92.92±4.43	<0.001
Platelet (×10^3/μL)	242.27±55.05	249.66±56.60	235.83±52.82	<0.001
HbA1c (%)	5.75±0.69	5.70±0.58	5.79±0.76	<0.001
HBsAg-positive, no. (%)	3330.80 (4.53%)	2790.90 (4.31%)	539.90 (6.08%)	<0.001
Anti-HCV Ab-positive, no. (%)	818.90 (1.11%)	657.80 (1.02%)	161.10 (1.81%)	<0.001
H. pylori Ab-positive, no. (%)	41,899.00 (56.95%)	36,775.20 (56.85%)	5123.90 (57.69%)	0.160
Retinal Arteriolar Sclerosis, no. (%)				
Grade 0	60,085.00 (81.99%)	53,941.00 (83.38%)	6361.00 (71.62%)	
Grade 1	10,554.00 (14.40%)	8661.00 (13.39%)	1956.00 (22.02%)	
Grade 2	2338.00 (3.19%)	1840.00 (2.84%)	504.00 (5.67%)	<0.001
Grade 3	296.00 (0.40%)	239.00 (0.37%)	58.00 (0.65%)	
Grade 4	14.00 (0.02%)	11.00 (0.02%)	3.00 (0.03%)	

Ab = antibody; ALP = alkaline phosphatase ; ALT = alanine transaminase; AST = aspartate transaminase; BMI = body mass index; BUN = blood urea nitrogen; Cr = creatinine; DM = diabetes mellitus; GGT = gamma-glutamyl transferase; HBsAg = hepatitis B surface antigen; HCV = hepatitis C; Hb = hemoglobin;

HbA1c = glycosylated hemoglobin; HDL = high-density lipoprotein; *H.pylori* = *Helicobacter pylori*; Hs-CRP = high-sensitivity c-reactive protein; HTN = hypertension; KRW = South Korean won; MCV = mean corpuscular volume ; P = inorganic phosphate; TG = triglyceride; UA = uric acid; WBC = white blood count. [1]No age-related macular degeneration vs overall age-related macular degeneration

Table S3. Characteristics of Normal and Early/intermediate Age-Related Macular Degeneration Participants before and after Multiple Imputation.

Variables	Before Multiple Imputation			After Multiple Imputation		
	**No Age-Related Macular Degeneration (n = 64,692)**[1]	**Early/intermediate Age-Related Macular Degeneration (n = 8735)**[1]	**P Value**	**No Age-Related Macular Degeneration (n = 64,692)** [1]	**Early/intermediate Age-Related Macular Degeneration (n = 8735)**[1]	**P Value**
Age group, no. (%)						
30-39 yrs	15,227 (23.54%)	598 (6.85%)		15,227 (23.54%)	598 (6.85%)	
40-49 yrs	23,073 (35.67%)	2234 (25.58%)		23,073 (35.67%)	2234 (25.58%)	
50-59 yrs	17,804 (27.52%)	3133 (35.87%)	<0.001	17,804 (27.52%)	3133 (35.87%)	<0.001
60-69 yrs	7243 (11.20%)	2123 (24.30%)		7243 (11.20%)	2123 (24.30%)	
≥70 yrs	1345 (2.08%)	647 (7.41%)		1345 (2.08%)	647 (7.41%)	
Gender, no. (%)						
Female	31,065 (48.02%)	3159 (36.16%)	<0.001	33,627 (51.98%)	5576 (63.84%)	<0.001
Male	33,627 (51.98%)	5576 (63.84%)		31065 (48.02%)	3159 (36.16%)	
Smoking, no. (%)						
Never	33,829 (54.19%)	3990 (47.33%)		35,059 (54.19%)	4156 (47.58%)	
Former	14,998 (24.03%)	2549 (30.24%)	<0.001	15,550 (24.04%)	2625 (30.05%)	<0.001
Current	13,597 (21.78%)	1891 (22.43%)		14,083 (21.77%)	1954 (22.37%)	
Drinking, no. (%)						
Never	17,980 (28.94%)	2373 (28.56%)		18,797 (29.03%)	2497 (28.59%)	
Former	1917 (3.09%)	268 (3.23%)	0.640	1997 (3.08%)	281 (3.22%)	0.586
Current	42,223 (67.97%)	5667 (68.21%)		43,958 (67.89%)	5957 (68.20%)	
Active lifestyle, no. (%)	41,289 (64.90%)	6038 (70.36%)	<0.001	41,996 (64.92%)	6138 (70.27%)	<0.001
DM, no. (%)	6048 (9.35%)	1285 (14.71%)	<0.001	6049 (9.35%)	1285 (14.71%)	<0.001
HTN, no. (%)	16,366 (25.30%)	3292 (37.70%)	<0.001	16,368 (25.30%)	3293 (37.70%)	<0.001
Dyslipidemia, no. (%)	24,498 (37.87%)	3650 (41.80%)	<0.001	24,501 (37.87%)	3650 (41.80%)	<0.001
Cardiovascular diseases, no. (%)	2143 (3.32%)	430 (4.93%)	<0.001	2146 (3.32%)	430 (4.93%)	<0.001
Cerebrovascular diseases, no. (%)	278 (0.43%)	62 (0.71%)	0.001	279 (0.43%)	62 (0.71%)	0.001

Cancer, no. (%)	1647 (2.55%)	280 (3.21%)	<0.001	1647 (2.55%)	280 (3.21%)	<0.001
Income, no. (%)						
<KRW 3,000,000	9103 (15.32%)	1644 (20.77%)	<0.001	9957 (15.39%)	1776 (20.33%)	<0.001
KRW 3,000,000- 5,000,000	13,088 (22.02%)	1623 (20.51%)		14,243 (22.02%)	1803 (20.64%)	
KRW 5,000,000- 10,000,000	21,225 (35.72%)	2586 (32.68%)		23,082 (35.68%)	2875 (32.91%)	
≥KRW 10,000,000	16,011 (26.94%)	2061 (26.04%)		17,410 (26.91%)	2281 (26.11%)	
Education level, no. (%)						
<Middle school graduate	7659 (12.07%)	1507 (17.68%)	<0.001	7818 (12.08%)	1534 (17.56%)	<0.001
Middle school graduate	11,517 (18.16%)	1832 (21.49%)		11,748 (18.16%)	1870 (21.41%)	
High school graduate	30,055 (47.38%)	3549 (41.63%)		30,650 (47.38%)	3647 (41.75%)	
≥College/university graduate	14,199 (22.39%)	1637 (19.20%)		14,476 (22.38%)	1684 (19.28%)	
Height (cm)						
Female	158.12±5.35	156.42±5.43	<0.001	158.14±5.37	156.45±5.47	<0.001
Male	164.56±8.30	164.59±8.36	0.714	164.55±8.30	164.59±8.36	0.745
BMI (kg/m²)						
Female	22.30±2.91	23.02±2.97	<0.001	22.30±2.91	23.02±2.97	<0.001
Male	23.46±3.05	23.92±2.86	<0.001	23.46±3.05	23.92±2.86	<0.001
Blood laboratory parameters						
Corrected calcium (mg/dL)	9.48±0.49	9.48±0.48	0.300	9.48±0.49	9.48±0.48	0.298
P (mg/dL)	3.66±0.58	3.64±0.62	0.042	3.66±0.58	3.64±0.62	0.043
BUN mg/dl (mg/dL)	13.56±3.61	14.39±3.69	<0.001	13.56±3.61	14.39±3.69	<0.001
Cr (mg/dL)	1.00±0.21	1.03±0.19	<0.001	1.00±0.21	1.03±0.19	<0.001
AST (U/L)	24.21±15.68	26.05±15.16	<0.001	24.21±15.68	26.04±15.15	<0.001
ALT (U/L)	25.90±25.10	27.62±23.38	<0.001	25.890±25.10	27.62±23.37	<0.001
ALP (U/L)	61.69±19.85	65.12±20.08	<0.001	61.68±19.85	65.11±20.08	<0.001
GGT (U/L)	34.14±43.27	38.83±47.01	<0.001	34.16±43.26	38.85±46.97	<0.001
Total bilirubin (mg/dL)	1.07±0.45	1.09±0.43	0.001	1.07±0.45	1.09±0.43	<0.001
UA (mg/dL)	5.53±1.43	5.68±1.46	<0.001	5.53±1.43	5.68±1.46	<0.001
Total cholesterol (mg/dL)	195.38±34.47	197.24±34.58	<0.001	195.38±34.47	197.23±34.58	<0.001
HDL (mg/dL)	54.35±13.43	53.14±13.19	<0.001	54.35±13.43	53.14±13.19	<0.001

TG (mg/dL)	114.51±77.27	119.80±86.02	<0.001	114.52±77.26	119.83±86.00	<0.001
Total albumin (g/dL)	4.38±0.26	4.35±0.27	<0.001	4.38±0.26	4.35±0.27	<0.001
Hs-CRP (mg/dL)	0.14±0.43	0.17±0.61	<0.001	0.19±0.43	0.20±0.59	0.032
Hb (g/dL)	14.31±1.63	14.54±1.54	<0.001	14.31±1.63	14.54±1.54	<0.001
WBC (x 10^3/μL)	5.75±1.68	5.79±1.66	0.018	5.75±1.68	5.79±1.66	0.019
MCV (fL)	92.24±4.90	93.17±4.72	<0.001	92.24±4.90	93.17±4.72	<0.001
Platelet (x 10^3/μL)	243.08±54.93	236.34±55.50	<0.001	243.08±54.93	236.37±55.50	<0.001
HbA1c (%)	5.73±0.68	5.85±0.75	<0.001	5.73±0.68	5.85±0.75	<0.001
HBsAg-positive, no. (%)	2787 (4.31%)	536 (6.15%)	<0.001	2791 (4.59%)	537 (6.15%)	<0.001
Anti-HCV Ab-positive, no. (%)	657 (1.02%)	157 (1.80%)	<0.001	658 (1.02%)	157 (1.80%)	<0.001
H. pylori Ab-positive, no. (%)	32,392 (56.85%)	4487 (57.84%)	0.099	36,775 (56.85%)	5048 (57.79%)	0.095
Retinal Arteriolar Sclerosis (n, %)						
No	53,941 (83.38%)	6274 (71.83%)		53,941 (83.38%)	6274 (71.83%)	
Low	8661 (13.39%)	1923 (22.01%)	<0.001	8661 (13.39%)	1923 (22.01%)	<0.001
High	2090 (3.23%)	538 (6.16%)		2090 (3.23%)	538 (6.16%)	

Ab = antibody; ALP = alkaline phosphatase ; ALT = alanine transaminase; AST = aspartate transaminase; BMI = body mass index; BUN = blood urea nitrogen; Cr = creatinine; DM = diabetes mellitus; GGT = gamma-glutamyl transferase; HBsAg = hepatitis B surface antigen; HCV = hepatitis C; Hb = hemoglobin; HbA1c = glycosylated hemoglobin; HDL = high-density lipoprotein; *H.pylori* = *Helicobacter pylori*; Hs-CRP = high-sensitivity c-reactive protein; HTN = hypertension; KRW = South Korean won; MCV = mean corpuscular volume ; P = inorganic phosphate; TG = triglyceride; UA = uric acid; WBC = white blood count.

Table S4. Logistic Regression Analyses for Associations between and Early/intermediate Age-Related Macular Degeneration before Multiple Imputation.

Variables	Univariable Analysis[2]		Multivariable Model[2]		
	**Odds Ratio (95% Confidence Interval)**	**P Value**	**Odds Ratio (95% Confidence Interval)**	**Original P Value**	**Benjamini-Hochberg–corrected P Value**
Age group, no. (%)					
30-39 yrs	1 (reference)		1 (reference)		
40-49 yrs	2.47 (2.25-2.70)	<0.001	2.25 (2.00-2.52)	<0.001	<0.001
50-59 yrs	4.48 (4.10-4.90)	<0.001	3.74 (3.32-4.21)	<0.001	<0.001
60-69 yrs	7.46 (6.79-8.21)	<0.001	5.56 (4.88-6.32)	<0.001	<0.001
≥70 yrs	12.25 (10.82-13.87)	<0.001	8.48 (7.16-10.05)	<0.001	<0.001
Gender, no. (%)					
Female	1 (reference)		1 (reference)		
Male	1.63 (1.56-1.71)	<0.001	1.93 (1.74-2.14)	<0.001	<0.001
Smoking, no. (%)					
Never	1 (reference)		1 (reference)		
Former	1.44 (1.37-1.52)	<0.001	0.98 (0.91-1.06)	0.634	0.750
Current	1.18 (1.11-1.25)	<0.001	1.06 (0.98-1.15)	0.152	0.319
Drinking, no. (%)					
Never	1 (reference)				
Former	1.06 (0.93-1.21)	0.403			
Current	1.02 (0.97-1.07)	0.519			
Active lifestyle, no. (%)	1.28 (1.22-1.35)	<0.001	0.99 (0.93-1.05)	0.653	0.750
DM, no. (%)	1.67 (1.57-1.79)	<0.001	0.97 (0.87-1.08)	0.550	0.750
HTN, no. (%)	1.79 (1.71-1.87)	<0.001	1.12 (1.05-1.19)	<0.001	0.002
Dyslipidemia, no. (%)	1.18 (1.13-1.23)	<0.001	0.94 (0.88-1.00)	0.036	0.113
Cardiovascular diseases, no. (%)	1.51 (1.36-1.68)	<0.001	0.98 (0.86-1.11)	0.714	0.785
Cerebrovascular diseases, no. (%)	1.66 (1.26-2.18)	<0.001	1.02 (0.74-1.41)	0.915	0.959
Cancer, no. (%)	1.27 (1.12-1.44)	<0.001	1.18 (1.00-1.38)	0.051	0.140
Income, no. (%)					
<KRW 3,000,000	1 (reference)		1 (reference)		
KRW 3,000,000- 5,000,000	0.69 (0.64-0.74)	<0.001	0.96 (0.88-1.06)	0.413	0.613

KRW 5,000,000- 10,000,000	0.68 (0.63-0.72)	<0.001	0.96 (0.87-1.05)	0.332	0.562
≥KRW 10,000,000	0.71 (0.67-0.76)	<0.001	0.93 (0.84-1.02)	0.113	0.249
Education level, no. (%)					
<Middle school graduate	1 (reference)		1 (reference)		
Middle school graduate	0.81 (0.75-0.87)	<0.001	1.03 (0.93-1.13)	0.590	0.750
High school graduate	0.60 (0.56-0.64)	<0.001	0.88 (0.80-0.96)	0.007	0.024
≥College/university graduate	0.59 (0.54-0.63)	<0.001	0.80 (0.72-0.89)	<0.001	<0.001
Height (cm)	1.00 (1.00-1.00)	0.714			
BMI (kg/m^2)	1.05 (1.04-1.06)	<0.001	1.01 (1.00-1.02)	0.226	0.410
Blood laboratory parameters					
Corrected calcium (mg/dL)	0.98 (0.93-1.02)	0.305			
P (mg/dL)	0.96 (0.92-1.00)	0.032	1.01 (0.97-1.06)	0.549	0.750
BUN mg/dl (mg/dL)	1.06 (1.05-1.07)	<0.001	1.01 (1.00-1.02)	0.066	0.160
Cr (mg/dL)	2.00 (1.80-2.22)	<0.001	0.83 (0.69-1.00)	0.045	0.132
AST (U/L)	1.01 (1.00-1.01)	<0.001	1.00 (1.00-1.01)	0.175	0.350
ALT (U/L)	1.00 (1.00-1.00)	<0.001	1.00 (1.00-1.00)	0.413	0.613
ALP (U/L)	1.01 (1.01-1.01)	<0.001	1.00 (1.00-1.00)	0.418	0.613
GGT (U/L)	1.00 (1.00-1.00)	<0.001	1.00 (1.00-1.00)	0.652	0.750
Total bilirubin (mg/dL)	1.09 (1.04-1.14)	0.001	1.02 (0.95-1.08)	0.634	0.750
UA (mg/dL)	1.07 (1.06-1.09)	<0.001	0.96 (0.94-0.99)	0.003	0.011
Total cholesterol (mg/dL)	1.00 (1.00-1.00)	<0.001	1.00 (1.00-1.00)	0.995	0.995
HDL (mg/dL)	0.99 (0.99-1.00)	<0.001	1.00 (1.00-1.01)	0.058	0.150
TG (mg/dL)	1.00 (1.00-1.00)	<0.001	1.00 (1.00-1.00)	0.233	0.410
Total albumin (g/dL)	0.66 (0.61-0.72)	<0.001	0.99 (0.88-1.11)	0.852	0.914
Hs-CRP (mg/dL)	1.13 (1.08-1.17)	<0.001	1.02 (0.97-1.08)	0.399	0.613
Hb (g/dL)	1.10 (1.08-1.11)	<0.001	1.00 (0.97-1.03)	0.952	0.974
WBC (x 10^3/μL)	1.02 (1.00-1.03)	0.018	1.00 (0.98-1.01)	0.659	0.750
MCV (fL)	1.04 (1.04-1.05)	<0.001	1.01 (1.00-1.02)	0.001	0.004
Platelet (x 10^3/μL)	1.00 (1.00-1.00)	<0.001	1.00 (1.00-1.00)	0.233	0.410
HbA1c (%)	1.23 (1.20-1.26)	<0.001	1.01 (0.96-1.06)	0.665	0.750
HBsAg-positive	1.45 (1.32-1.60)	<0.001	1.42 (1.26-1.59)	<0.001	<0.001
Anti-HCV Ab-positive	1.78 (1.50-2.13)	<0.001	1.22 (0.99-1.50)	0.069	0.160
H.pylori Ab-positive	1.04 (0.99-1.09)	0.098			

Retinal Arteriolar Sclerosis (n, %)					
No	1 (reference)		1 (reference)		
Low	1.91 (1.81-2.02)	<0.001	1.32 (1.24-1.42)	<0.001	<0.001
High	2.21 (2.01-2.44)	<0.001	1.30 (1.15-1.47)	<0.001	<0.001

Ab = antibody; ALP = alkaline phosphatase ; ALT = alanine transaminase; AST = aspartate transaminase; BMI = body mass index; BUN = blood urea nitrogen; Cr = creatinine; DM = diabetes mellitus; GGT = gamma-glutamyl transferase; HBsAg = hepatitis B surface antigen; HCV = hepatitis C; Hb = hemoglobin; HbA1c = glycosylated hemoglobin; HDL = high-density lipoprotein; *H.pylori* = *Helicobacter pylori*; Hs-CRP = high-sensitivity c-reactive protein; HTN = hypertension; KRW = South Korean won; MCV = mean corpuscular volume ; P = inorganic phosphate; TG = triglyceride; UA = uric acid; WBC = white blood count. [1]Results are based on imputed data. [2]Results are based on nonimputed data.

Table S5. Characteristics of Normal and Advanced Age-Related Macular Degeneration Participants before and after Multiple Imputation.

	Before Multiple Imputation			After Multiple Imputation		
Variables	**No Age-Related Macular Degeneration (n = 64,692)***	**Advanced Age-Related Macular Degeneration (n = 147)***	**P Value**	**No Age-Related Macular Degeneration (n = 64,692)***	**Advanced Age-Related Macular Degeneration (n = 147)***	**P Value**
Age group, no. (%)						
30-39 yrs	15,227 (23.54%)	5 (3.40%)		15,227 (23.54%)	5 (3.40%)	
40-49 yrs	23,073 (35.67%)	24 (16.33%)		23,073 (35.67%)	24 (16.33%)	
50-59 yrs	17,804 (27.52%)	30 (20.41%)	<0.001	17,804 (27.52%)	30 (20.41%)	<0.001
60-69 yrs	7243 (11.20%)	58 (39.46%)		7243 (11.20%)	58 (39.46%)	
≥70 yrs	1345 (2.08%)	30 (20.41%)		1345 (2.08%)	30 (20.41%)	
Gender, no. (%)						
Female	31,065 (48.02%)	42 (28.57%)	<0.001	33,627 (51.98%)	42 (28.57%)	<0.001
Male	33,627 (51.98%)	105 (71.43%)		31,065 (48.02%)	105 (71.43%)	
Smoking, no. (%)						
Never	33,829 (54.19%)	61 (41.78%)		35,059 (54.19%)	43 (41.50%)	
Former	14,998 (24.03%)	56 (38.36%)	<0.001	15,550 (24.04%)	7 (38.10%)	<0.001
Current	13,597 (21.78%)	29 (19.86%)		14,083 (21.77%)	97 (20.41%)	
Drinking, no. (%)						
Never	17,980 (28.94%)	42 (29.37%)		18737 (28.96%)	43 (29.25%)	
Former	1917 (3.09%)	7 (4.90%)	0.444	1997 (3.09%)	7 (4.76%)	0.492
Current	42,223 (67.97%)	94 (65.73%)		43958 (67.95%)	97 (65.99%)	
Active lifestyle, no. (%)	41,289 (64.90%)	106 (73.10%)	0.046	41,997 (64.92%)	107 (72.79%)	0.048
DM, no. (%)	6048 (9.35%)	27 (18.37%)	0.001	6049 (9.35%)	27 (18.37%)	0.001
HTN, no. (%)	16,366 (25.30%)	77 (52.40%)	0.00	16,368 (25.30%)	77 (52.40%)	0.00
Dyslipidemia, no. (%)	24,498 (37.87%)	60 (40.82%)	0.497	24,500 (37.87%)	60 (40.82%)	0.497
Cardiovascular diseases, no. (%)	2143 (3.32%)	12 (8.22%)	0.004	2146 (3.32%)	12 (8.22%)	0.004

Cerebrovascular diseases, no. (%)	278 (0.43%)	2 (1.37%)	0.133	279 (0.43%)	2 (1.37%)	0.134
Cancer, no. (%)	1647 (2.55%)	6 (4.08%)	0.283	1647 (2.55%)	6 (4.08%)	0.283
Income, no. (%)						
<KRW 3,000,000	9103 (15.32%)	38 (28.36%)	0.001	9957 (15.39%)	41 (28.89%)	<0.001
KRW 3,000,000- 5,000,000	13,088 (22.02%)	24 (1791%)		14,243 (22.02%)	27 (18.37%)	
KRW 5,000,000- 10,000,000	21,225 (35.72%)	43 (32.09%)		23,082 (35.68%)	47 (31.97%)	
≥KRW 10,000,000	16,011 (26.94%)	29 (21.64%)		17,410 (26.91%)	32 (21.77%)	
Education level, no. (%)						
<Middle school graduate	7659 (12.07%)	33 (22.76%)	<0.001	7818 (12.08%)	33 (22.45%)	<0.001
Middle school graduate	11,517 (18.16%)	34 (23.45%)		11,748 (18.16%)	35 (23.81%)	
High school graduate	30,055 (47.38%)	49 (33.79%)		30,650 (47.38%)	50 (34.01%)	
≥College/university graduate	14,199 (22.39%)	29 (20.00%)		14,476 (22.38%)	29 (19.73%)	
Height (cm)						
Female	158.12±5.35	155.63±6.19	0.003	158.14±5.72	155.68±5.33	0.003
Male	170.49±5.71	168.09±5.27	<0.001	170.48±5.72	168.06±5.33	<0.001
BMI (kg/m^2)						
Female	22.29±2.91	23.40±3.55	0.014	22.29±2.76	23.40±2.82	0.014
Male	24.15±2.83	24.18±2.83	0.158	24.53±2.76	24.15±2.81	0.163
Blood laboratory parameters						
Corrected calcium (mg/dL)	9.48±0.49	9.41±0.53	0.077	9.48±0.49	9.41±0.48	0.078
P (mg/dL)	3.66±0.58	3.76±0.78	0.032	3.66±0.58	3.76±0.62	0.032
BUN mg/dl (mg/dL)	13.56±3.61	15.74±4.83	<0.001	13.56±3.61	15.74±3.71	<0.001
Cr (mg/dL)	1.00±0.21	1.07±0.22	<0.001	1.00±0.21	1.07±0.19	<0.001
AST (U/L)	24.21±15.68	25.93±17.33	0.185	24.21±15.67	25.93±15.19	0.203
ALT (U/L)	25.90±25.10	25.47±15.70	0.836	25.89±25.09	25.47±23.27	0.835
ALP (U/L)	61.69±19.85	66.32±20.60	0.005	61.68±19.85	66.32±20.08	0.005
GGT (U/L)	34.14±43.27	50.29±164.84	<0.001	34.16±43.26	50.29±51.17	<0.001
Total bilirubin (mg/dL)	1.07±0.45	1.06±0.50	0.763	1.07±0.45	1.06±0.43	0.764
UA (mg/dL)	5.53±1.43	5.91±1.44	0.001	5.53±1.43	5.91±1.46	0.001
Total cholesterol (mg/dL)	195.38±34.47	194.02±33.53	0.633	195.38±34.47	194.02±34.56	0.632

HDL (mg/dL)	54.35±13.43	54.44±14.72	0.941	54.35±13.43	54.44±13.22	0.941
TG (mg/dL)	114.51±77.27	109.14±59.82	0.400	114.52±77.26	109.14±88.64	0.402
Total albumin (g/dL)	4.38±0.26	4.25±0.28	<0.001	4.38±0.26	4.25±0.27	<0.001
Hs-CRP (mg/dL)	0.14±0.43	0.23±0.61	0.069	0.19±0.43	0.24±0.59	0.256
Hb (g/dL)	14.31±1.63	14.18±1.93	0.962	14.31±1.63	14.18±1.55	0.362
WBC (x 10^3/μL)	5.75±1.68	5.80±2.03	0.770	5.75±1.68	5.80±1.67	0.768
MCV (fL)	92.24±4.90	93.14±6.89	0.026	92.24±4.90	93.14±4.76	0.026
Platelet (x 10^3/μL)	243.08±54.93	235.03±58.38	0.076	243.08±54.93	235.03±55.54	0.076
HbA1c (%)	5.73±0.68	5.98±1.00	0.004	5.73±0.68	5.97±0.75	0.003
HBsAg-positive, no. (%)	2787 (4.31%)	3 (2.04%)	0.222	2791 (4.31%)	3 (2.04%)	0.222
Anti-HCV Ab-positive, no. (%)	657 (1.02%)	4 (2.72%)	0.065	658 (1.02%)	4 (2.72%)	0.065
H. pylori Ab-positive, no. (%)	32,392 (56.85%)	66 (50.00%)	0.157	36,775 (56.85%)	75 (51.02%)	0.157
Retinal Arteriolar Sclerosis (n, %)						
No	53,941 (83.38%)	87 (59.18%)		53,941 (83.38%)	87 (59.18%)	
Low	8661 (13.39%)	33 (22.45%)	<0.001	8661 (13.39%)	33 (22.45%)	<0.001
High	2090 (3.23%)	27 (18.37%)		2090 (3.23%)	27 (18.37%)	

Ab = antibody; ALP = alkaline phosphatase ; ALT = alanine transaminase; AST = aspartate transaminase; BMI = body mass index; BUN = blood urea nitrogen; Cr = creatinine; DM = diabetes mellitus; GGT = gamma-glutamyl transferase; HBsAg = hepatitis B surface antigen; HCV = hepatitis C; Hb = hemoglobin; HbA1c = glycosylated hemoglobin; HDL = high-density lipoprotein; *H.pylori* = *Helicobacter pylori*; Hs-CRP = high-sensitivity c-reactive protein; HTN = hypertension; KRW = South Korean won; MCV = mean corpuscular volume ; P = inorganic phosphate; TG = triglyceride; UA = uric acid; WBC = white blood count.

Table S6. Logistic Regression Analyses for Associations between and Advanced Age-Related Macular Degeneration before Multiple Imputation.

Variables	Univariable Analysis[2]		Multivariable Model[2]		
	**Odds Ratio (95% Confidence Interval)**	**P Value**	**Odds Ratio (95% Confidence Interval)**	**Original P Value**	**Benjamini-Hochberg–corrected P Value**
Age group, no. (%)					
30-39 yrs	1 (reference)		1 (reference)		
40-49 yrs	3.17 (1.21-8.30)	0.019	4.23 (0.98-18.30)	0.053	0.182
50-59 yrs	5.13 (1.99-13.23)	0.001	6.12 (1.43-26.25)	0.015	0.083
60-69 yrs	24.39 (9.78-60.82)	<0.001	20.60 (4.79-88.50)	<0.001	0.001
≥70 yrs	67.93 (26.31-175.36)	<0.001	53.11 (11.80-239.07)	<0.001	<0.001
Gender, no. (%)					
Female	1 (reference)		1 (reference)		
Male	2.31 (1.61-3.30)	<0.001	2.84 (1.59-5.06)	<0.001	0.003
Smoking, no. (%)					
Never	1 (reference)		1 (reference)		
Former	2.07 (1.44-2.98)	<0.001	1.16 (0.72-1.86)	0.547	0.752
Current	1.18 (0.76-1.84)	0.457	1.08 (0.61-1.89)	0.800	0.903
Drinking, no. (%)					
Never	1 (reference)				
Former	1.56 (0.70-3.48)	0.275			
Current	0.95 (0.66-1.37)	0.796			
Active lifestyle, no. (%)	1.47 (1.02-2.12)	0.040	1.03 (0.68-1.57)	0.876	0.903
DM, no. (%)	2.18 (1.44-3.32)	<0.001	0.68 (0.37-1.27)	0.229	0.540
HTN, no. (%)	3.25 (2.35-4.49)	<0.001	1.47 (0.99-2.16)	0.055	0.182
Dyslipidemia, no. (%)	1.13 (0.81-1.57)	0.463			
Cardiovascular diseases, no. (%)	2.61 (1.44-4.72)	0.001	1.22 (0.65-2.32)	0.534	0.752
Cerebrovascular diseases, no. (%)	3.20 (0.79-13.00)	0.103			
Cancer, no. (%)	1.63 (0.72-3.69)	0.243			
Income, no. (%)					

<KRW 3,000,000	1 (reference)		1 (reference)		
KRW 3,000,000- 5,000,000	0.44 (0.26-0.73)	0.002	0.82 (0.47-1.43)	0.476	0.752
KRW 5,000,000- 10,000,000	0.49 (0.31-0.75)	0.001	0.92 (0.54-1.58)	0.766	0.903
≥KRW 10,000,000	0.43 (0.27-0.70)	0.001	0.83 (0.46-1.48)	0.519	0.752
Education level, no. (%)					
<Middle school graduate	1 (reference)		1 (reference)		
Middle school graduate	0.69 (0.42-1.11)	0.122	1.00 (0.57-1.76)	0.994	0.994
High school graduate	0.38 (0.24-0.59)	<0.001	0.75 (0.42-1.32)	0.314	0.648
≥College/university graduate	0.47 (0.29-0.78)	0.003	0.92 (0.49-1.75)	0.807	0.903
Height (cm)	1.00 (0.98-1.02)	0.959			
BMI (kg/m^2)	1.05 (1.00-1.11)	0.059	0.95 (0.89-1.01)	0.122	0.336
Blood laboratory parameters					
Corrected calcium (mg/dL)	0.74 (0.53-1.03)	0.077	0.94 (0.58-1.51)	0.788	0.903
P (mg/dL)	1.30 (1.03-1.65)	0.028	1.34 (1.09-1.66)	0.007	0.046
BUN mg/dl (mg/dL)	1.08 (1.06-1.10)	<0.001	1.04 (0.99-1.09)	0.105	0.315
Cr (mg/dL)	1.71 (1.31-2.24)	<0.001	0.53 (0.19-1.43)	0.210	0.533
AST (U/L)	1.00 (1.00-1.01)	0.191			
ALT (U/L)	1.00 (0.99-1.01)	0.834			
ALP (U/L)	1.00 (1.00-1.01)	0.005	1.00 (0.99-1.01)	0.520	0.752
GGT (U/L)	1.00 (1.00-1.00)	<0.001	1.00 (1.00-1.00)	0.042	0.173
Total bilirubin (mg/dL)	0.94 (0.65-1.38)	0.762			
UA (mg/dL)	1.12 (1.05-1.20)	<0.001	1.01 (0.87-1.18)	0.872	0.903
Total cholesterol (mg/dL)	1.00 (0.99-1.00)	0.633			
HDL (mg/dL)	1.00 (0.99-1.01)	0.941			
TG (mg/dL)	1.00 (1.00-1.00)	0.397			
Total albumin (g/dL)	0.18 (0.10-0.31)	<0.001	0.39 (0.18-0.85)	0.018	0.085
Hs-CRP (mg/dL)	1.23 (1.04-1.45)	0.015	0.98 (0.78-1.23)	0.846	0.903
Hb (g/dL)	0.96 (0.87-1.05)	0.362			
WBC (x $10^3/\mu L$)	1.02 (0.93-1.11)	0.722			
MCV (fL)	1.04 (1.01-1.06)	0.012	0.99 (0.95-1.03)	0.538	0.752
Platelet (x $10^3/\mu L$)	1.00 (0.99-1.00)	0.075	1.00 (1.00-1.00)	0.770	0.903
HbA1c (%)	1.34 (1.17-1.52)	<0.001	1.13 (0.87-1.45)	0.360	0.699
HBsAg-positive	0.46 (0.15-1.45)	0.186			

Anti-HCV Ab-positive	2.72 (1.01-7.38)	0.049	1.45 (0.51-4.10)	0.489	0.752
H.pylori Ab-positive	0.76 (0.54-1.07)	0.114			
Retinal Arteriolar Sclerosis (n, %)					
No	1 (reference)		1 (reference)		
Low	2.36 (1.58-3.53)	<0.001	1.27 (0.81-1.98)	0.293	0.623
High	8.01 (5.19-12.36)	<0.001	3.05 (1.83-5.07)	<0.001	<0.001

Ab = antibody; ALP = alkaline phosphatase ; ALT = alanine transaminase; AST = aspartate transaminase; BMI = body mass index; BUN = blood urea nitrogen; Cr = creatinine; DM = diabetes mellitus; GGT = gamma-glutamyl transferase; HBsAg = hepatitis B surface antigen; HCV = hepatitis C; Hb = hemoglobin; HbA1c = glycosylated hemoglobin; HDL = high-density lipoprotein; *H.pylori* = *Helicobacter pylori*; Hs-CRP = high-sensitivity c-reactive protein; HTN = hypertension; KRW = South Korean won; MCV = mean corpuscular volume ; P = inorganic phosphate; TG = triglyceride; UA = uric acid; WBC = white blood count. [1]Results are based on imputed data. [2]Results are based on nonimputed data.
